# Emerging Paradigms in Inflammatory Disease Management: Exploring Bioactive Compounds and the Gut Microbiota

**DOI:** 10.3390/brainsci13081226

**Published:** 2023-08-21

**Authors:** Tarek Benameur, Chiara Porro, Mohammed-Elfatih Twfieg, Nassima Benameur, Maria Antonietta Panaro, Francesca Martina Filannino, Abeir Hasan

**Affiliations:** 1 Department of Biomedical Sciences, College of Medicine, King Faisal University, Al-Ahsa 31982, Saudi Arabia; 2 Department of Clinical and Experimental Medicine, University of Foggia, 71122 Foggia, Italy; 3 Faculty of Exact Sciences and Sciences of Nature and Life, Research Laboratory of Civil Engineering, Hydraulics, Sustainable Development and Environment (LARGHYDE), Mohamed Khider University, Biskra 07000, Algeria; 4 Department of Biosciences, Biotechnologies and Environment, University of Bari, 70125 Bari, Italy

**Keywords:** inflammation, inflammatory mediators, bioactive compounds, anti-inflammatory, inflammatory bowel disease (IBD), colorectal cancer (CRC), gut microbiota–brain axis GMBA, gut microbiota

## Abstract

The human gut microbiota is a complex ecosystem of mutualistic microorganisms that play a critical role in maintaining human health through their individual interactions and with the host. The normal gastrointestinal microbiota plays a specific physiological function in host immunomodulation, nutrient metabolism, vitamin synthesis, xenobiotic and drug metabolism, maintenance of structural and functional integrity of the gut mucosal barrier, and protection against various pathogens. Inflammation is the innate immune response of living tissues to injury and damage caused by infections, physical and chemical trauma, immunological factors, and genetic derangements. Most diseases are associated with an underlying inflammatory process, with inflammation mediated through the contribution of active immune cells. Current strategies to control inflammatory pathways include pharmaceutical drugs, lifestyle, and dietary changes. However, this remains insufficient. Bioactive compounds (BCs) are nutritional constituents found in small quantities in food and plant extracts that provide numerous health benefits beyond their nutritional value. BCs are known for their antioxidant, antimicrobial, anticarcinogenic, anti-metabolic syndrome, and anti-inflammatory properties. Bioactive compounds have been shown to reduce the destructive effect of inflammation on tissues by inhibiting or modulating the effects of inflammatory mediators, offering hope for patients suffering from chronic inflammatory disorders like atherosclerosis, arthritis, inflammatory bowel diseases, and neurodegenerative diseases. The aim of the present review is to summarise the role of natural bioactive compounds in modulating inflammation and protecting human health, for their safety to preserve gut microbiota and improve their physiology and behaviour.

## 1. Introduction

The human gut microbiota refers to a diverse community of mutualistic microorganisms inhabiting the human gastrointestinal (GI) tract. This includes bacteria, fungi, viruses, archaea, and protozoa [1]. The gut microbiota represents a complex ecosystem and is thought to be the most important in preserving human health through their interaction with each other and the host [2]. The normal GI microbiota was shown to have a specific physiological function in host immunomodulation, nutrient metabolism, vitamin synthesis, xenobiotic and drug metabolism, maintenance of structural and functional integrity of the gut mucosal barrier, and protection against various pathogens. Indeed, this central regulator role is also referred to as the ‘second brain’ given its importance in preserving host physiology and homeostasis [3]. Significant interest in gut microbiota research has rapidly evolved over the past decade. Increasing evidence has associated gut microbiota with many human diseases associated with inflammation, including inflammatory bowel disease (IBD) [4,5].

Inflammation is a term used to describe the innate immune response of living tissues to injury and damage caused by exposure to various harmful conditions, including infections, physical and chemical trauma, immunological factors and genetic derangements [6,7]. Most of the diseases that affect humans are found to be associated with an underlying inflammatory process. Acute inflammation serves as a protective response that helps the body fight off infections and repair tissue damage. However, chronic inflammation occurs when the inflammatory response persists over an extended period, leading to tissue damage and dysfunction in various organs and systems within the body. This chronic inflammation is implicated in the pathogenesis of various diseases [8,9].

The primary aim of inflammation is to reduce the impact of tissue injury and prepare for healing and repair. Inflammation is mediated through the contribution of various active immune cells (e.g., macrophages, neutrophils, lymphocytes, endothelial cells and platelets) that produce chemical molecules and cytokines in addition to plasma proteins [6]. Despite the beneficial effect of inflammation, noticeable tissue damage may accompany this process, and it is more prominent in the case of chronic inflammation [10].

The current strategies used to control the inflammatory process include pharmaceutical drugs, lifestyle, and dietary changes.

However, due to the difficulty in controlling the pathophysiological process associated with chronic inflammation-associated diseases, the investigation of the therapeutic and preventive potentials of bioactive compounds (BCs) is gaining significant research interest. Thus, understanding their role provides an opportunity for a new trend in different pathologies with a particular focus on inflammation [11].

BCs are nutritional constituents found in small quantities in food and plant extracts that are able to provide numerous health benefits beyond their nutritional value. Many BCs are derived from plants, and their extracts are considered excellent candidates. Their pharmacological properties have been intensively studied to investigate their role in relation to human health, given their antioxidant, antimicrobial, anticarcinogenic, anti-metabolic syndrome, and anti-inflammatory properties [12,13,14,15]. There are many different types of BCs, each with its own unique set of properties and potential health benefits. Some examples include polyphenols, flavonoids, carotenoids, tannins and alkaloids. The most frequently studied are polyphenols, especially flavonoids and anthocyanins found in plant parts and have various physiological and functional benefits to maintain normal health [16]. High consumption of foods rich in BCs with antioxidant properties, such as vitamins, phytochemicals, and mainly phenolic compounds such as flavonoids and carotenoids, reduces the pro-inflammatory state, metabolic disorders, and oxidative stress [17,18].

Furthermore, polyphenols are a diverse group of BCs known for their antioxidant and anti-inflammatory properties and have been shown to modulate the human gut microbiota positively. Green tea, berries (blueberries, strawberries, raspberries), cocoa, red wine, apples, onions, and curcumin from turmeric are good examples of polyphenol sources [19,20].

Resveratrol is a polyphenol found in grapes, red wine, and berries. Research has indicated that resveratrol can modulate gut microbiota and promote the growth of beneficial bacteria. A recent study has reported that resveratrol supplementation increased the abundance of *Lactobacillus* and *Bifidobacterium* species in mice [21]. Another subclass of polyphenols is flavonoids which are widely distributed in fruits, vegetables, and herbs. This category can be found in citrus fruits (such as lemons, oranges), soybeans, parsley, and *Ginkgo biloba*. They possess anti-inflammatory, antioxidant, and prebiotic effects [22,23].

Quercetin is an abundant flavonoid in various fruits and vegetables. It has been investigated for its potential prebiotic effects on gut microbiota. Research has shown that consuming quercetin can promote the growth of beneficial bacteria, including *Bifidobacterium* and *Akkermansia* while reducing harmful bacteria in the gut [24,25,26].

Terpenes and terpenoids are the main BCs of essential oils (EOs). EOs are highly concentrated and volatile liquids derived from various plant parts. EOs contain BCs, primarily terpenes and terpenoids, which exhibit diverse biological activities such as anticancer, antimicrobial, antioxidant, antiallergic and anti-inflammatory properties. For instance, they can be found in plants like oregano, thyme, lavender, citrus peel, and medicinal herbs such as Echinacea and ginseng (e.g., Echinacea, ginseng) [27].

Alkaloids are nitrogen-containing compounds with at least one nitrogen atom in a heterocyclic ring structure, mainly found in plants. Some alkaloids have been investigated for their effects on gut microbiota and potential health benefits. They often have significant pharmacological activities and potential impact on gut microbiota. Notable examples of alkaloids were found in various sources, including caffeine from coffee beans, theanine from green tea, and codeine from the opium poppy. Researchers are increasingly interested in these compounds and understanding their potential role in promoting gut health and overall well-being.

Caffeine consumption can influence the gut microbiota composition, specifically increasing beneficial Bifidobacterium and Lactobacillus species [9]. These alterations in the gut microbiota could potentially impact gut health positively. Additionally, theanine supplementation in mice led to an increased level of beneficial bacteria, such as Lactobacillus and Bifidobacterium, in the gut [28].

Dietary fibre is also considered a BC with significant effects on gut microbiota. It serves as a prebiotic by providing nourishment for beneficial gut microbiota and by promoting their growth and activity. They are not fully digestible by human enzymes but serve as substrates for fermentation by gut microbiota, leading to the production of SCFAs. SCFAs play a critical role in maintaining gut health, including providing energy to colonocytes, regulating inflammation and stimulating mucus production and strengthening the gut barrier [29,30].

The relationship between dietary fibre and gut microbiota has been extensively studied, and there is substantial evidence supporting their positive impact on gut health. Dietary fibre, particularly certain types such as inulin, oligosaccharides, and resistant starch, resist digestion in the upper gastrointestinal tract and reach the colon largely intact. In the colon, it becomes a source of nutrition for beneficial bacteria, such as *Bifidobacteria* and *Lactobacilli*. These bacteria ferment dietary fibre, producing SCFAs as by-products. Recent evidence demonstrated that inulin was able to increase the levels of Bifidobacterium and *Faecalibacterium prausnitzii* in the gut, which is associated with gut health [31,32].

Sulfur-containing compounds have emerged as promising bioactive agents found in certain vegetables and herbs and have been recognised for their potential benefits on gut health. These compounds are abundant in garlic, onions, and cruciferous vegetables such as broccoli, cauliflower, and kale. Allicin is one of the main sulfur-containing compounds in these foods. These compounds have been studied for their antimicrobial properties and their ability to support the growth of beneficial gut bacteria. Additionally, sulfur-containing compounds, particularly those in cruciferous vegetables, can undergo enzymatic breakdown in the gut, leading to the formation of bioactive metabolites with potential health-promoting effects [33].

**Figure 1:**
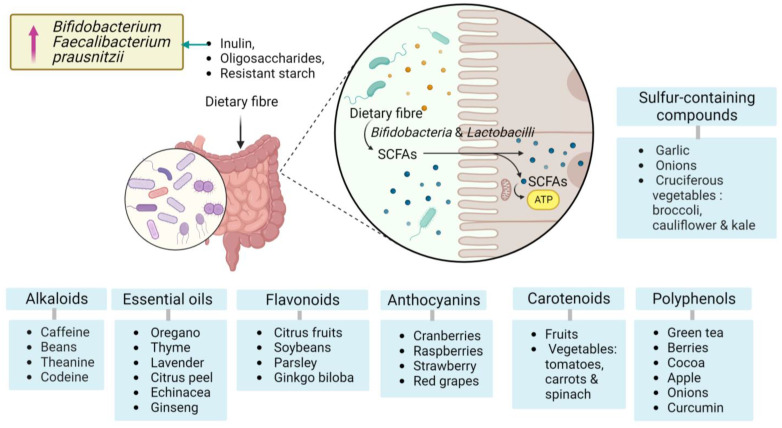
Categories of bioactive compounds for gut microbiota with potential sources. Polyphenols, carotenoids, anthocyanins, flavonoids, essential oils, alkaloids, sulfur-containing compounds, dietary fibres and their effects on *Bifidobacterium* and *Faecalibacterium* species. Created with BioRender.com, accessed on 8 August 2023.

Figure 1 illustrates some categories of BCs for gut microbiota and their potential sources.

Current research in the field of anti-inflammatory agents focuses on minimising the associated destructive effect of inflammation on diverse tissues by blocking or modulating the effects of inflammatory mediators utilising bioactive compounds. Many studies were promising and offered hope for many patients, especially those suffering from debilitating chronic inflammatory disorders such as atherosclerosis, arthritis, inflammatory bowel diseases, and neurodegenerative diseases [12,34]. In this review, we will have a glance at the latest update to highlight the different aspects of using bioactive compounds as a potential therapeutic approach in the modulation, management, and prevention of various inflammatory diseases, with a special focus on inflammatory bowel disease (IBD), colorectal cancer (CRC), and neurodegenerative diseases. We will also discuss the importance of the human gut microbiota–brain axis (GMBA) in controlling inflammation via the BCs.

Table 1 demonstrates the taxonomy of the targeted microbiota by BCs in the regulation of inflammation.

Despite the significant efforts made in this area, it is still important to identify and investigate the role of natural bioactive compounds that could selectively target and modulate inflammation and affect the GMBA in order to protect human health.

## 2. Pathophysiology of Inflammation

The word “inflammation” originates from the Latin word “inflammatio”, which means fire. It is the body’s protective response against injury and is clinically characterised by five cardinal signs: redness, swelling, pain, warmth/heat, and loss of function [39,40,41]. These clinical signs result from specific cellular and molecular processes activated during the inflammatory response. Redness and warmth/heat occur due to an increased blood flow, while swelling is caused by fluid accumulation. The pain is a consequence of both swelling and the release of substances that generate nerve signals [39].

Inflammation is triggered when host cells detect conserved structures on pathogens known as pathogen-associated molecular patterns (PAMPs) [42] or endogenous stress signals known as danger-associated molecular patterns (DAMPs) through pattern-recognition receptors (PRRs), which are predominantly expressed on myeloid cells, such as macrophages, monocytes, neutrophils, and dendritic cells [40]. Activation of these immune cells leads to the production of pro-inflammatory cytokines and chemokines [42].

TNF and IL-1β are potent pro-inflammatory cytokines that act through autocrine and paracrine mechanisms. They stimulate acute-phase protein production in the liver, activate platelets, and induce fever, fatigue, and anorexia. Additionally, these cytokines promote endothelial cell activation, increasing vascular permeability and immune cells migration into tissues at the site of infection. However, this activation can also lead to harmful systemic effects, such as capillary leakage, vasodilation, and hypotension [40].

Chemokines recruit additional immune cells, such as neutrophils, to the infection site, where they play a significant role in phagocytosis and pathogens’ elimination [43,44,45]. Neutrophils are activated by the cytokine IFN-γ, while IL-22 acts on epithelial cells, stimulating the production of antimicrobial peptides (AMPs), including defensins [40,46].

Upon activation, monocytes and neutrophils in the bloodstream trigger the release of prostaglandins. Prostaglandins mediate the signs and symptoms of illness, such as somnolence, fatigue, and fever, by acting on the hypothalamus. Additionally, inflammatory mediators in the circulation activate the complement system, which mediates microbial opsonisation and killing, producing inflammatory peptides such as C3a and C5a12 [40].

After eliminating the inflammatory trigger, it is important to control the inflammatory response and restore tissue homeostasis. Failure to resolve inflammation can lead to chronic inflammatory diseases such as arthritis, colitis, or asthma, with permanent tissue damage and an increased risk of cancer, cardiovascular disease, and osteoporosis [47].

The resolution of inflammation involves three key processes. The first process is the cessation of neutrophil influx, controlled by pro-resolving lipid mediators (resolvins). Resolution involves a class-switch from producing pro-inflammatory mediators such as PGE2 and LTB4 to pro-resolving lipid mediators such as prostaglandin D2, lipoxin A4 (LXA4), resolvin E1 (RvE1), and maresin-1. These mediators can block neutrophil recruitment by downregulating their chemokine receptors, such as CXCR2, making them unresponsive to neutrophil-activating substances like LTB4, KC, and complement factors [47].

The second process is neutrophil apoptosis induced by death ligands such as TRAIL or FasL, produced by macrophages or by TGFβ, produced by regulatory T cells during the resolution phase of inflammation. Macrophages rapidly engulf apoptotic neutrophils through efferocytosis [47]. The third process encompasses alterations in macrophage function. During the immune response, monocyte-derived macrophages contribute to cytokine production and pathogen clearance. However, in the inflammatory phase, they acquire important anti-inflammatory and pro-resolution functions. They remove apoptotic cells, release pro-resolving lipids, express anti-inflammatory receptors such as TGF-R2 and FPR2, and synthesise increased concentrations of immune regulatory intracellular messengers such as cAMP [47].

If the inflammatory inducer is not eliminated by the acute inflammatory response or persists due to unrepaired tissue damage, chronic infections, or other reasons, the resolution phase may not be appropriately induced, leading to a chronic inflammatory state. This localised chronic inflammation can cause different types of tissue remodelling, like granuloma formation in persistent infections or respiratory epithelial tissue remodelling in asthma induced by allergens [48]. The severity and duration of chronic inflammation vary depending on the injury cause and the body’s repair abilities [49].

Recent research highlights the significant role of the microbiota in regulating the inflammatory process. A balanced microbial community supports immune homeostasis, promoting anti-inflammatory responses and maintaining gut barrier integrity. However, dysbiosis, characterised by an imbalance in microbial composition, can lead to an inappropriate immune activation, triggering or exacerbating inflammatory conditions. Specific microbial species, such as segmented filamentous bacteria, have been found to induce pro-inflammatory responses. Understanding the complex interactions between gut microbiota and inflammation remains critical to open new avenues for therapeutic interventions targeting various inflammatory diseases [50].

## 3. Inflammation Pathogenesis

The process of inflammation involves a highly coordinated network of mediators and cellular events. The inflammatory phagocytic cells produce intracellular Reactive Oxygen Species (ROS) within their phagolysosome to dismantle the phagocytosed organisms or particles through lipids and protein oxidation. ROS contribute significantly to tissue damage associated with inflammation. This oxidative stress is regulated by antioxidant enzymes like catalase, superoxide dismutase and glutathione peroxidase. Nitric oxide (NO), in three forms (eNO, nNO, and iNO), also plays a critical role in the inflammatory pathway [6]. The understanding of inflammation and its role in pathogenesis has been reinforced by the development of sensitive biomarkers. Besides the inflammatory mediators discussed earlier, other inflammatory biomarkers are involved in inflammation pathogenesis, including the formation of DNA adducts, acute-phase proteins like C-reactive protein (CRP), prostaglandins, Cyclooxygenase (COX)-related metabolites, major immune cell types, inflammation-related growth factors and transcription factors [51].

Uncontrolled chronic inflammation possesses a remarkable capacity to facilitate nearly all essential cellular and molecular capabilities necessary for tumorigenesis. The exact mechanisms by which inflammatory cells promote neoplastic transformation are not completely understood.

However, it has been observed that hepatocellular carcinoma and gastric adenocarcinoma associated with chronic viral infection and *H. pylori* infection, respectively, develop as a consequence of persistent inflammatory changes that precede neoplasia. Additionally, IBDs are also associated with an increased incidence of colorectal cancer [52].

NF-κB activation plays a significant role in the pathogenesis of various inflammatory conditions, including atherosclerosis and viral infections, driven by stimulated immune cells like lymphocytes. Activated NF-κB modulates the transcription of genes related to the inflammatory response and immune mediators, including cytokine genes [6,53].

Cytokines such as TNF, IFN-γ, IL-1, and IL-6 are crucial participants in inflammation produced by inflammatory cells. They attract leukocytes, stimulate acute-phase protein production, and increase body temperature through hypothalamic thermoregulation [7].

Recent evidence highlights the crucial role of gut microbiota in regulating immune responses and inflammation throughout various body systems [54,55,56,57]. The gut microbiota exerts potent immune modulatory effects on the host, influencing the balance between pro-inflammatory and anti-inflammatory responses.

Dysbiosis is associated with inflammatory conditions, both locally in the gut and systemically. Changes in the gut microbiota have been associated with IBDs, such as Crohn’s disease and ulcerative colitis, as well as systemic conditions like rheumatoid arthritis, inflammation associated with obesity, and neuroinflammation.

Gut microbiota has been implicated in either promoting or dampening inflammation. For example, some species belonging to the *Bacteroides* and *Ruminococcus genera* have been associated with pro-inflammatory effects, while others, like *Faecalibacterium prausnitzii* and *Akkermansia muciniphila*, have shown anti-inflammatory properties.

Advancements in metagenomic sequencing and other high-throughput techniques have provided deeper insights into the gut microbiota’s role in inflammation, enabling the identification of specific microbial signatures associated with different inflammatory diseases.

Understanding the interactions between gut microbiota and inflammation holds great promise for developing targeted and personalised therapeutic approaches. Modulating the gut microbiota through probiotics, prebiotics, postbiotics, and precision dietary interventions has shown potential in managing inflammatory disorders and restoring immune homeostasis [54,55,56,57].

## 4. Gut Microbiota and Its Biological Functions

The human gut microbiota is a complex, dynamic, and spatially heterogeneous ecosystem inhabited by a community of microorganisms. It comprises a trillion microorganisms such as bacteria, viruses, fungi, yeasts, archaea, and bacteriophages, with the most important bacterial species including Actinobacteria, Bacteroidetes, Firmicutes, and Proteobacteria. The Bacteroidetes and Firmicutes account for 90% of the GI microbiota [58,59,60]. Given that the GI tract is divided anatomically and functionally into three segments: the stomach, small and large intestine, it is important to emphasise that the physicochemical barrier and the distinct microenvironment of each compartment lead to the growth of specific GI microbiota [61].

Starting to develop from birth and continuing until reaching a stable status [62]. Its composition and function can be affected by a variety of factors, including environmental factors, diet, drug use, age, genetics and lifestyle throughout the human lifetime. This microbiota differs between healthy individuals [63]. The gut microbiota lives with the host in a symbiotic relationship and plays a fundamental role in the host’s physiology and pathophysiology [64]. A complex network of interactions involving the exchange of metabolic, immune, and neuroendocrine signals was shown to regulate and stabilise the symbiotic relationship between the microbiota and the host.

Some of the well-documented biological functions of the microbiota include digestion and absorption of nutrients and the production of vitamins, which are essential for human health, such as vitamin K, B12, and folic acid. Additionally, microbiota helps to protect against the colonisation of the intestine by exogenous pathogens and potentially harmful indigenous microorganisms.

There is growing evidence that microbiota is involved in the development and the modulation of the host immune responses, influencing multiple host organs [65,66,67,68,69]. Furthermore, the microbiota plays a vital role in the regulation of metabolism through the production of hormones and regulation of the inflammatory process. Microbiota was also described as the virtual endocrine organ [70].

Gut microbiota and their metabolites influence the release of CCK, PYY, GLP-1, GIP, and 5-HT, which are produced and secreted by the enteroendocrine cells in the mucosal lining [71].

When we compare the cell composition, genetic diversity, and metabolic capacity, the host should be considered a multispecies hybrid organism consisting of host cells and microbial cells which operate in a dynamic and symbiotic manner [72].

Recent research findings have shown the association of dysbiosis with several health conditions, including diabetes, obesity, cardiovascular diseases, GI disorders, cancer, and neurodegenerative diseases such as Parkinson’s disease and Alzheimer’s disease. Dysbiosis is defined as a functional and compositional alteration in the microbiota caused by a combination of environmental and host-related factors that disrupt the microbial ecosystem to a degree that exceeds its resilience and resistance capabilities. Dysbiosis is also known as a stable microbial community condition that contributes to the aetiology, diagnosis, or treatment of a wide range of diseases [73,74].

The controversial role of gut microbiota in regulating human metabolism has prompted researchers to investigate the role of these microorganisms in relation to metabolic pathways, particularly those associated with nutrients. In addition to catabolic and biological transformation functions, gut microbiota creates small bioactive molecules that facilitate interactions with hosts and contribute to the neurohumoral axis that connects the intestine to other body parts [75].

Another aspect attracting researchers’ interest relates to microbiota diversity. The diverse microbial community that lives in the human gut has a large metabolic repertoire that differs from but complements the activity of mammalian enzymes in the liver and gut mucosa, and it contains functions that are required for host digestion [4]. Recent studies on animal gut microbiota may be a potential source of novel bioactive molecules. However, this requires further investigation [76].

The gut microbiota generates a wide range of metabolites by breaking down indigestible carbohydrates. For example, SCFAs produced by the fermentation of dietary fibre have been shown to have anti-inflammatory effects and may protect against colorectal cancer. Peptidoglycan and lipopolysaccharides (LPS) are complex macromolecules required for bacterial integrity.

The presence of bacteria and their metabolites in the gut can influence hormone secretion. The gut hormone release mediated by microbes is an important component of microbial regulation of host metabolism. Dietary or pharmaceutical therapies that alter the gut microbiota present an excellent therapeutic strategy for treating human metabolic diseases.

## 5. Interplay between the ‘Gut Microbiota–Brain Axis’ and Inflammation

The gut and the brain are in a dynamic bidirectional communication involving the central and the enteric nervous systems (ENS), associating the cognitive and emotional centres of the brain with the peripheral intestinal functions [77,78,79,80]. Recent research findings have shown that the gut–brain axis is modulated by the gut microbiota, together forming the gut microbiota–brain axis (GMBA). The GMBA refers to the network of connections between several biological systems that allows bidirectional communication between the GI microbiota and the brain. GMBA is critical to maintaining homeostasis of the GI, CNS, and microbial systems [81,82,83]. This bidirectional neurohumoral communication system involves both direct and indirect signalling through neuronal, chemical, and immune mediators that enable the brain to influence GI functions, such as motility, secretion, and mucin production, and modulation of cytokine release by cells of the mucosal immune system [84,85,86]. Considering the impact of multiple biological systems, this suggests an interrelationship between the different signalling pathways and mechanisms that mediate various aspects of disease pathogenesis. Despite the progress that has been made in this regard, further investigations are needed to elucidate these mechanisms.

The gut–brain axis has been modelled by a variety of animal models and human research. As described previously, the factors that contribute to GMBA balance include diet, stress, sleep, exercise, social interaction, happiness, neurodegenerative disorders, environmental factors, drug use, mode of delivery, genetics and epigenetics, cognitive behaviour, and food intake [1].

The majority of the information on host–microbiota interactions, and hence the available data in the literature, are acquired from studies on animal models in which researchers can efficiently control the test animals’ environment. The autonomic nervous system (e.g., ENS and the vagus nerve), the neuroendocrine system, the hypothalamic–pituitary–adrenal axis (HPA), the immune system, and metabolic pathways are all involved in communication [82]. The gut microbiota produces neurotransmitters, such as GABA, amino acids (e.g., tryptophan, tyramine), noradrenaline, dopamine, and serotonin (5-hydroxytryptamine (5-HT).

These metabolites can cross the portal circulation and interact with the host immune system, regulate metabolism, and/or activate local neuronal cells of the ENS and vagus nerve afferent pathways that transmit signals directly to the brain. The gut microbiota can also impair the integrity of the gut barrier, which restricts the transit of signalling molecules from the intestine lumen to the lamina propria, which contains immune cells and the terminal ends of ENS neurons, or to portal circulation.

Anxiety, autism spectrum disorder, and depression are all neuropsychiatric illnesses that can impair gut barrier integrity. Stress can stimulate the HPA axis response, which involves hypothalamic neurons that release hormones such as corticotropin receptor hormone (CRH) into the brain or the portal circulation, triggering the synthesis and release of cortisol. Cortisol was shown to regulate the neuroimmune signalling responses, which in turn, can affect the intestinal barrier integrity. Immune mediators, stress hormones, and CNS neurotransmitters can activate ENS neurons and vagus nerve afferent pathways, which may change the gut environment and alter the microbiota composition [82,83,84,85,86].

Recent evidence has demonstrated that GMBA plays an essential role in regulating CNS neuroinflammation and behaviour. Taking into consideration the putative relationship among gut microbiota, neural function, and behaviour, in this section, the role of GMBA with regard to inflammation is discussed in detail.

One of the most innovative therapeutic approaches having a positive impact on GMBA is the faecal microbiota transfer from a healthy individual. Indeed, the transfer of the faecal microbiota from a healthy, screened donor to a recipient is known as faecal microbiota transplantation (FMT), also known as “faeces transplantation”, “human intestinal microbiota transfer”, and “faecal bacteriotherapy” [87].

From a therapeutic point of view, FMT has grown in popularity in order to repair imbalances, modify and restore damaged microbiota.

The administration of FMT exhibited a suppressive effect on the activation of Iba1-positive microglia cells and Glial Fibrillary Acidic Protein (GFAP)-positive astrocyte cells. This finding underscores the capability of FMT to modulate gut microbiota dysbiosis, thereby ameliorating intestinal tract inflammation, intestinal mucosal disruption, and neuroinflammation induced by chronic unpredictable stress in rats. Moreover, FMT demonstrated the capacity to regulate serotonin concentrations, which are primarily biosynthesised within the intestinal tract and consequently alleviated depressive-like behaviour [88,89]. To date, FMT has predominantly been employed in clinical settings for the management of recurrent or refractory *Clostridioides difficile* infections (rCDI), yielding success rates of up to 90%. Furthermore, FMT has been proven to surpass antibiotic therapy in the treatment of CDI [87,88,89,90].

Consistent with prior research findings, the GMBA is not only crucial for maintaining overall health but also appears to have a growing involvement in various neurological disorders, such as PD, AD, autism spectrum disorder, and major depressive disorders [91,92]. Another study supports the therapeutic potential of FMT administration with a particular focus on the rotenone-induced PD mouse model through the GMBA. The authors demonstrated that dysbiosis of the gut microbiota induced by rotenone led to GI functional impairment and compromised behavioural performance in PD mice. Furthermore, 16S RNA sequencing revealed an increase in the bacterial genera *Akkermansia* and *Desulfovibrio* in rotenone-induced mouse faeces. In contrast, FMT therapy effectively restored the gut microbial ecosystem, alleviating GI dysfunctions and motor impairments in PD mice. Further investigation showed that FMT treatment reduced systemic inflammation by mitigating intestinal inflammation and preserving the integrity of the intestinal barrier. Subsequently, FMT therapy improved the integrity of the blood–brain barrier (BBB) and inhibited neuroinflammation in the substantia nigra (SN), resulting in less damage to dopaminergic neurons. Mechanistic studies also revealed that FMT treatment decreased levels of LPS in the colon, serum, and SN, thereby inhibiting the TLR4/MyD88/NFk-B signalling pathway and its downstream pro-inflammatory products in both the SN and the colon [93]. As further discussed below, inflammatory responses are not only associated with GI disorders such as IBS, IBD, and CRC but also contribute to metabolic, reproductive, autoimmune, cardiovascular, and neurodegenerative diseases [94,95,96,97,98,99]. The intestinal barrier, known as the intestinal mucosal or epithelial barrier (IEB), consists of a mucus layer, an epithelial barrier, and a gut vascular barrier. It plays a crucial role in maintaining health and preserving diseases. IEB acts as a selectively permeable barrier, facilitating nutrient absorption while preventing the entry of harmful substances and pathogens present in the intestines [100,101]. The gut microbiota directly influences the intestinal mucosal barrier (IEB), which serves as the body’s first line of defence against pathogens’ invasion. The gut microbiota directly influences the development and differentiation of intestinal epithelial cells (ECs), tight junction protein production, and mucosal permeability, thereby preserving the integrity of the IEB [102].

Any impairment in the composition of gut microbiota can result in the alteration of IEB and intercellular junction functions, which increase intestinal permeability and the inflammatory mediator transport. IEB function can be impaired by changes in the composition of the gut microbiota, which can also increase intestinal permeability and the transport of inflammatory mediators. The gut lamina propria responds to signals from bacterial and metabolic components by inducing an inflammatory response, which connects the gut microbiota to chronic inflammation in the organism, which plays an essential role in the pathogenesis of several diseases [97,103]. The innate immune system, which responds immediately to the gut microbiota, is found in the intestine and comprises the natural killer cells, Paneth cells, macrophages, neutrophils, mast cells and dendritic cells [104,105]. These cells and epithelial cells have pattern recognition receptors (PRRs), such as NLR, TLR, retinoic acid-inducible gene (RIG)-I-like receptor (RLR), C-type lectin receptor (CLR), and deletion 2 (AIM2)-like receptor (ALR) [100]. Pathogen-associated molecular patterns (PAMPs) or microbial metabolites associated with the gut microbiota are recognised by these receptor families. PAMPs, which include lipopolysaccharide (LPS), peptidoglycan (PGN), lipoteichoic acid (LTA), and flagellin (FLG), are conserved structural components in microorganisms that activate the innate immune system within the intestine by specifically binding to certain receptors. This process modulates the interactions between microbiota and the host while also influencing immunological tolerance [106,107].

## 6. Gut Microbiota–Brain Axis and Neurodegenerative Diseases

First, the GM can create and release neurotransmitters and neurotoxins such as D-lactate, ammonia, acetylcholine, SCFAs, 5HT, and acetylcholine. All these molecules are transported by the circulatory system before crossing the BBB to modulate neural activity. Second, the ENS is connected to the CNS via the vagus nerve and the autonomic nervous system. When the ENS is activated, it receives signals from the GM, acts on intestinal cells, and controls the anti-inflammatory effects of the peripheral immune system. Finally, the GM is involved in modulating the immune system through the synthesis and release of pro-inflammatory cytokines, such as interleukin-1, interleukin-6, and tumour necrosis factor-alpha [108].

The gut–brain axis (GBA) is made up of the CNS, PNS, ENS, immune system, and endocrine systems, which work together to form a network for transmitting information between the gut and the brain. The GBA acts as the bidirectional link between the CNS and the endocrine system. It connects the brain’s thinking and feeling centres to the intestine’s peripheral functions, which are controlled by the endocrine and immune systems, intestinal epithelium, and GI. Thus, the GBA has an essential role in the bidirectional communication between the ENS and the CNS [108].

There is strong evidence that any alteration in these pathways is linked to neurodegenerative disorders. Due to gut dysbiosis, pathogenic microbial metabolites and pro-inflammatory mediators are overproduced, resulting in a leaky gut. Increased intestinal permeability and BBB disruptions are additional consequences of gut dysbiosis. Changes in the composition of the gut microbiota enhance gut barrier permeability and immunological activation, both of which lead to systemic inflammation [109]. Gut bacteria regulate the differentiation and activity of immune cells in the stomach, periphery, and brain.

The BBB is highly selective under physiological conditions, blocking the passage of toxins and harmful biochemical signals. Dysregulation of BBB permeability allows the infiltration of immune signalling molecules, leukocytes and bacterial and pro-inflammatory elements. This is a key factor in triggering neuroinflammation and neurodegeneration [110]. Several investigations have revealed that CNS glial cells are activated by bacteria-derived stimuli and develop pathological features such as protein fibrils and inclusions. The continuous exposure of CNS resident cells to inflammatory stimuli induces a continuous glial over-response [111]. There is evidence that persistent microglial overstimulation impairs the ability to respond to pathological signals. Thus, in most neurodegenerative diseases, activated CNS glial cells have a direct role in the pathogenesis and progression of the disease [110].

### 6.1. Alterations in the Gut Microbiota in Parkinson’s Disease

Pathological Parkinson’s disease (PD) is the loss of dopaminergic neurons in the striatum and substantia nigra, where clusters of α-synuclein accumulate to form Lewy bodies. Ageing is undoubtedly the major risk factor for PD, with changes in energy metabolism, oxidative stress, inflammation, etc., contributing to the onset of neuronal loss [112].

However, the mechanisms underlying the development of PD remain poorly understood. Recently, the GI tract, GM, and gut–brain crosstalk have been highlighted as potential mechanisms underlying PD progression.

Many studies have compared patients with PD with healthy controls to investigate variations in the gut microbiota. The role of GI microorganisms in triggering intestinal inflammation is still widely studied. The study by Braak Del and Tredici proposes that abnormal αSyn buildup begins in the gut and propagates prion-like to the brain via the vagus nerve. Indeed, alpha-Syn inclusions are seen early in the ENS and the glossopharyngeal and vagal nerves [113]. Individuals with PD have alterations in particular microbial populations, which are implicated in PD pathogenesis. For example, *Helicobacter pylori* and *Ralstonia* in the GI are significantly increased in PD patients [114].

Recent meta-analysis studies have focused on the importance of the increased population of twenty-two bacteria identified in Parkinson’s disease, including the Akkermansia genus, *Verrucomicrobiaceae* family, *Rikenellaceae* family, *Lactobacillus* genus, Lactobacillaceae family, *Bifidobacterium* genus, Bifidobacteriaceae family, Proteobacteria phylum, Alistipes genus, *Actinobacteria* phylum, *Verrucomicrobia* phylum, *Enterobacteriaceae* family, *Streptococcus* genus, and *Ruminococcaceae* family that would be increased in the disease. Other bacteria decrease in Parkinson’s disease: *Roseburia* genus, *Lachnospiraceae* family, *Faecalibacterium* genus, *Prevotellaceae* family, *Prevotella* genus, *Blautia* genus, *Bacteroidetes* phylum, and *Fusicatenibacter* genus [115,116,117].

Moreover, a recent investigation conducted by Murros et al. specifically focuses on elucidating the role of the *Desulfovibrionaceae* family. The study revealed that members of this bacterial family adhere to the intestinal wall while producing lipopolysaccharide (LPS) and hydrogen sulphide, a chemical considered neurotoxic in high concentrations. The elevated levels of hydrogen sulphide prompt the aggregation of alpha-synuclein, resulting in intestinal neurodegeneration. The mechanisms contributing to the initiation of intestinal neurodegeneration encompass several factors: disruption of the gut’s mucus layer (*Akkermansia muciniphila, Bifidobacterium, Desulfovibrionaceae*), disturbance in the production of short-chain fatty acids (SCFA), increased production of pro-inflammatory cytokines (TNFα, IL-1, IL-17, IFN-γ and IL-6), and the production of LPS in the gut [118,119]. Notably, several recent studies suggest that exposure to LPS-producing bacteria could be a driving force behind alpha-synucleinopathies. Hasegawa et al. investigated the intestinal microbiota in PD and healthy cohabitants, showing that PD patients exhibited a higher abundance of Lactobacillus compared to controls, whereas the Clostridium coccoides group and the Bacteroides fragilis group were lower in PD patients than in controls [120,121].

Additionally, another study by Sampson et al. demonstrates the essential role of gut microbiota in motor deficits, microglial activation, and α-Synuclein pathology. Through germ-free or antibiotic-depleted conditions, transgenic animals overexpressing human α-Synuclein exhibited reduced microglial activation, α-Synuclein inclusions and motor deficits compared to animals with complex microbiota [122,123].

Recent research has demonstrated that the gut microbiota actively promotes the full maturation and inflammatory potential of microglia by generating SCFAs [124].

SCFAs can cross the BBB or have peripheral effects, activating microglia via mechanisms that are currently unknown. SCFAs, which include acetic acid, propionic acid, and butyric acid, are bacterial fermentation products that have recently been demonstrated to be crucial for immune cell homeostasis in the colon. SCFAs can cross the intestinal mucosa into the systemic circulation, where they can affect immune regulation and CNS [124,125,126].

In support of the involvement of the gut microbiota in the aggregation and pathogenic spread of αSyn, the study by Grathwohl et al. provides evidence that DSS colitis triggers αSyn accumulation in the ENS of wild-type mice and in a human αSyn transgenic mouse model of PD. Furthermore, they show that chronic but transient DSS colitis in young αSyn transgenic mice leads to a markedly exacerbated accumulation of αSyn aggregates in the brain of aged mice [127]. Another study in 2019 by van Kessel et al. analysed the effect of levodopa-metabolising bacteria, particularly in the jejunum, where levodopa is absorbed. In fact, tyrosine decarboxylase (TDC) genes are encoded in the genome of several bacterial species in the genera Lactobacillus and Enterococcus. Although TDC is named for its ability to decarboxylate L-tyrosine to tyramine, it may also have the ability to decarboxylate levodopa to produce dopamine due to the high similarity of the chemical structures of these substrates. This suggests that the TDC activity of the gut microbiota may interfere with the availability of levodopa/decarboxylase inhibitors and, thus, the treatment of Parkinson’s patients [128].

### 6.2. Effects of Gut Microbiota on Alzheimer’s Disease

Alzheimer’s disease (AD) is a progressive neurodegenerative disease. The two histological hallmarks of AD are neurofibrillary tangles and extracellular β-amyloid peptide (Aβ) deposits within senile plaques in the CNS. Clinical studies have also investigated the composition of the gut microbiota in patients with AD. It has been seen that *Proteobacteria, Bifidobacterium*, and *Phascolarctobacterium* are significantly more abundant in patients with AD. Additionally, *Escherichia coli*-derived neurotoxins and *Proteobacteria* are correlated with AD neuropathology and increase the release of pro-inflammatory cytokines. Patients with the AD spectrum have an abnormally high abundance of *Proteobacteria*, a feature that has been suggested as a predictor of AD pathogenesis [129]. Hung et al. in 2022 proved that the genus *Bifidobacterium* is involved in the production of acetate and γ-aminobutyric acid, which have neuroprotective effects on the host. In addition, animal studies have shown that *Bifidobacterium* appears to attenuate the development of AD pathology. *Bifidobacterium* probiotics have also been reported to improve cognitive impairment in Alzheimer’s patients [108]. Recent studies have linked *H. pylori* infection to AD. Because the BBB restricts peptide transport from the periphery to the brain, BBB dysfunction may lead to the accumulation of peripheral Aβ in the brain and/or decreased clearance of brain Aβ. Park et al. hypothesise the mechanism by which H. pylori infection leads to BBB dysfunction. Chronic H. pylori infection increases gastric pH, causing atrophic gastritis and intestinal metaplasia. Homocysteine levels in the blood increase as the pH change reduces the absorption of vitamin B12 and folic acid. Homocysteine auto-oxidation produces hydrogen peroxide, which damages vascular endothelial cells, which compose the BBB. Subsequent BBB dysfunction and reduced blood flow caused by high blood homocysteine then lead to increased Aβ accumulation. In addition, *H. pylori* infection is associated with increased comorbid conditions, such as cardiovascular disease and diabetes mellitus, both of which can also be causes of BBB dysfunction [130].

A recent study conducted in 2022 provides a more comprehensive understanding of the metabolic pathways influenced by *H. pylori*. The research demonstrates that *H. pylori* induces increased intestinal permeability by activating the TLR4/Myd88 inflammatory pathway in a p53-dependent manner, thereby leading to metabolic dysfunction. Furthermore, the deficiency of p53 results in reduced bile acid concentrations, ultimately leading to enhanced colonisation of *H. pylori*. These findings collectively highlight the significant role of *H. pylori* in promoting metabolic dysfunction associated with AD-induced metabolic dysfunction [131].

### 6.3. Gut Changes in Huntington’s Disease

Huntington’s disease (HD) is an inherited neurodegenerative disorder characterised by a triad of motor, cognitive, and psychiatric impairment, as well as involuntary weight loss. HD is caused by the age-dependent penetrance of an expanded sequence of cytosine adenine guanine (CAG) repeats in the huntingtin gene. In addition to the cognitive, motor and neuropsychiatric symptoms that are thought to be related to changes in the brain, people with HD also experience a range of gastrointestinal disturbances, including diarrhoea, nutritional deficiencies, gastritis, and unintentional weight loss, which are recognised as clinical manifestations of HD.

There is some evidence suggesting that these disorders are a manifestation of gastrointestinal dysfunction [129]. Regarding the association between the GBA and HD, certain SCFAs and bioactive elements released by the GBA have been observed to influence the progression of HD. These substances primarily affect the biological functions of the GBA. Specifically, compounds such as tyrosine, IPA, 2-hydroxyphenylacetic acid, 3-hydroxyphenylacetic acid, and 4-hydroxyphenylacetic acid can lead to dysbiosis of the GBA by diet and BCs, while 5-HT, tyrosine, and these acids and hydroxyphenyl acetic acids can cause intestinal permeability [132,133]. Consequently, it is apparent that GM plays a role in maintaining both brain physiology and gut flora, and this has received particular attention from the scientific community. Although there are correlations established between host physiology and microbiota, it is important to highlight that causal relationships have not yet been established. Further research in this field is needed to gain a deeper understanding of the complex interplay between gut microbiota and neurodegenerative diseases.

## 7. The Interplay between Inflammatory Bowel Diseases and Human Gut Microbiota

IBD is a chronic inflammatory disease of the GI tract that is divided into Ulcerative colitis (UC) and Crohn’s disease (CD). These two disease entities vary in their histological morphology and site of involvement; they are characterised by GI tract inflammatory features that result from the interaction of the host immune response with some environmental and local factors in genetically liable persons. In addition to the intestinal tract, these diseases exhibit extraintestinal systemic clinical features [6].

The etiopathogenesis of IBD is not completely understood. Researchers proposed that the basis of this inflammatory disorder is the interaction between molecular alterations, the mucosal immune response, and the gut microbiota. This yields an altered host immunity against intestinal bacteria which elicits continuous inflammation. Various molecular alterations related to the pathogenesis of IBD were described. Aberrations involving various loci of the NOD2 gene were identified and proposed to be responsible for the weak immune response against bacteria linked to the development of CD. Besides that, autophagy genes ATG16L1 and IRGM mutations, NLRP3 inflammasome activation, unregulated activation of effector T-cells (Th1 and Th17) with the bulk release of inflammatory cytokines, TNF-α, IL-1, and IL-6, is markedly associated with increased risk of CD and play an important role in the pathogenesis. On the other hand, the incidence of UC was found to be increased with mutated mucosal barrier genes. Of more interest is the link between HLA genes and disease expression [134,135].

Defective host immune response to the gut microbiota and ineffective intestinal epithelial barriers greatly impact the development of IBD [136]. Consider this a prognostic factor for disease relapse. Intestinal mucosa exposed to long-term inflammation in IBD is at high risk of developing dysplasia and, later on, neoplasia in affected individuals [6]. In addition, numerous modifications in the viral community of IBD patients’ gut microbiota have been identified in recent studies.

Despite the fact that the functional significance of the altered bacteriophage profiles in IBD patients is unknown. Bacteriophages are viruses that parasitise and replicate within bacteria, and their integration into bacterial genomes may influence gene expression and function. Deep metagenomic sequencing of IBD patients’ mucosal and luminal samples demonstrated an increase in specific bacteriophage species. This reflects the significance of the interplay between human microbiota and IBD [137,138,139].

The cornerstone in the treatment of IBD is immune modulation to reduce inflammation and the associated tissue damage. In this respect, corticosteroids and Azathioprine induce and maintain remission in the course of Crohn’s disease, respectively, while the famous anti-inflammatory 5-aminosalicylic acid remains the gold standard in the treatment of Ulcerative colitis [134].

However, the long-term side effects and the effectiveness of the available treatment in the prevention of relapsing inflammatory episodes remain controversial. Emerging therapeutic approaches to modulating the microbiota are increasingly attracting researchers’ attention.

In the context of intestinal microbiota disorder, researchers have demonstrated some effective treatment methods for IBD through the improvement of intestinal microecology, including the use of prebiotics, probiotics, antibiotics, postbiotics, symbiotics, and FMT [140].

Taken together, these findings support the bidirectional model between IBD progression and changes in the microbiota community and functions.

## 8. The Role of Bioactive Compounds in Modulating Inflammatory Pathways Associated with Diseases

Part of the anti-inflammatory effects of some BCs, like flavonoids, are thought to be related to their ability to create antioxidant activity [141]. In addition to this, there is also evidence of the analgesic effect exhibited by these compounds using pain models, as revealed by [142]. Another point of importance is the antibacterial effect advocated to be exhibited by some bioactive compounds like Beta-Caryophyllene, as stated by Dickson et al. [143]. Bag et al. revealed the same result when they studied bioactive molecules found in essential oils of some spices for their antibacterial and antioxidant effects; they declared that some of these compounds, namely the coriander/cumin seed oil combination proved effective as antimicrobial and antioxidant [144]. More information in this regard is presented by Mahboubi et al. In their study, they reported the antimicrobial and antioxidant activities of flavonoid and phenolic compounds isolated from *S. striata* [145]. The involvement of BCs, specifically flavonoids, in modulating the transcription factor NF-κB and subsequently reducing inflammation biomarkers has captured the growing interest of researchers. This relationship has been extensively examined and validated through rigorous scientific investigation, affirming its significance in the field [146,147,148].

With regards to IBD management, bioactive compounds are studied extensively, accompanied by some observations postulated that consumption of plants and food rich in bioactive compounds can modulate the disease process in IBD. The antioxidant activity of polyphenols is considered to improve the inflammation induced in mice in a study by [149]. They noticed that the marked histological inflammatory features of colitis in the tested mice’s colon were markedly diminished with the use of (0.15 and 0.1 mg) phenolic extracts from grape pomace seeds.

Furthermore, Bitzer et al. studied soy protein concentrate based on its effects on the colonic mucosal barrier. They identified the redox activity of soy protein in vitro in alleviating induced colonic inflammation in mice. They assert that soy protein extract has an obvious role in lowering NLRP3 expression and caspase-1 activity, hence abating inflammation induced in the colon with the improvement of the mucosal barrier [150]. This is also supported by Liu et al. [148], who explored the therapeutic response of a flavonoid compound, Oroxindin, in mice with induced colitis. They revealed that Oroxindin prevents the activation of the NLRP3 inflammasome and TXNIP-dependent NF-κB activation, and hence believe that these BCs, found in the Chinese herb Huang-Qin, could be of emerging importance in treating IBD.

Panaxynol, a bioactive molecule extracted from American ginseng, was reported by Chaparala et al. to be effective in treating colitis in experimental mice; they postulated that this efficacy is correlated to its action on macrophage DNA leading to apoptosis. In addition, they noticed a reduction in the number of macrophages in inflamed colonic tissue treated with panaxynol in vitro [151].

Lee and Bae report further on the regulation and modulation of inflammation biomarkers. They studied, in vivo and in vitro, three polyphenols (Baicalin, Baicalein, and Wogonin) isolated from the Chinese herb Huang Qui for their anti-inflammatory effects. They stated that these compounds displayed variable degrees of reduction in vascular permeability, CAMs expression, and TNF-α in addition to downregulation of NF-κB. So, they considered them promising anti-inflammatory compounds [152].

Evaluation of the anticancer activity of bioactive compounds has been the aim of many studies carried out in vitro and in vivo. Clemente et al. supported this field with a study in which they tested the effect of protease inhibitors, rTI1B and rTI2B, extracted from recombinant (*Pisum sativum* L.) pea seeds, on the cells of adenocarcinoma of the colon, in vivo. They reported that these protease inhibitors have the ability to reduce the rate of growth of these malignant cells [153]. Another promising result stated that beta-carotene can suppress the COX-2 gene and enhance apoptosis in cells of adenocarcinoma of the colon [154].

**Figure 2:**
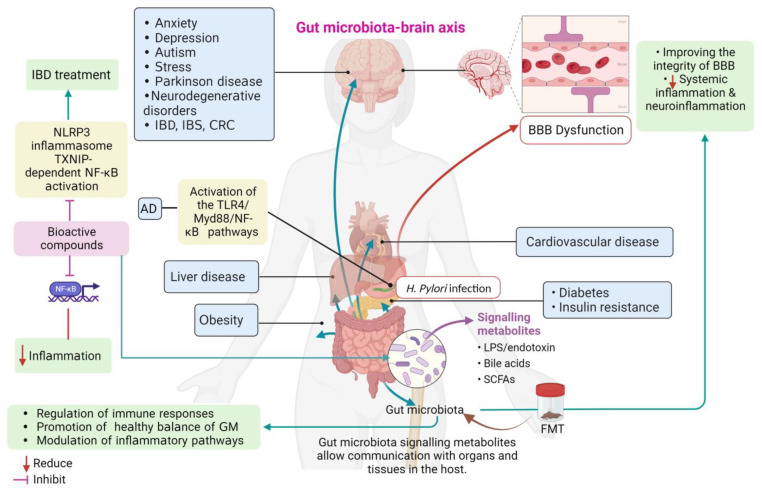
The role of bioactive compounds and Gut microbiota–brain axis (GMBA) in modulating inflammation associated with diseases. AD: Alzheimer’s disease; FMT: Faecal microbiota transplantation; IBD: Inflammatory bowel disease; IBS: irritable bowel syndrome; CRC: colorectal cancer; GM: gut microbiota; BBB: blood–brain barrier; SCFAs: Short-chain fatty acids; TXNIP: thioredoxin-interacting protein; NF-κB: nuclear factor-kappa B; NLRP3 (NLR Family Pyrin Domain Containing 3). Created with BioRender.com, accessed on 8 August 2023.

Figure 2 depicts some of the above-mentioned modulatory effects of bioactive compounds and GMBA on inflammation associated with diseases.

## 9. The Protective and Preventive Role of Bioactive Compounds against the Development of Inflammatory-Associated Diseases

Extensive work has been carried out by some researchers in order to find a way to halt the tissue damage that is associated with the inflammatory pathogenesis of some disorders like cardiovascular diseases, diabetes mellitus, and their complications. Compounds like polyphenols, through their antioxidant ability, significantly reduce the oxidative stress of tissue with inflammation, showing improvement in the oxidative stress index and are considered of value in the prevention and treatment of inflammatory disorders [155].

An interesting result was found when treating and preventing diabetes mellitus with gallic acid and *p*-coumaric acid. The apparent reduction of TNF-α, increased levels of PPARγ mRNA expression, and downturn of glycosylated haemoglobin and glucose levels in type 2 diabetic rats managed with these compounds were displayed and correlated with their anti-inflammatory and anti-diabetic effects [156].

Bioactive molecules are most studied for the prevention of cardiovascular disorders, specifically ischemic heart diseases. The antioxidant activity of polyphenols is found to reduce the level of low-density lipoprotein (LDL) oxidation and contributes to the reduction of the potential for developing ischemic heart disease [157]. This effect was also proven by the use of alpha-tocopherol as an antioxidant to prevent oxidation of LDL; in a randomised placebo-controlled single-blind study that revealed lower levels of LDL oxidation with the use of alpha-tocopherol at 6 and 12 weeks and, thence, possible lower risk of atherogenicity and its complications [158].

A recent study reported a protective effect on liver cells against acute injury caused by Diclofenac; they stated that *Opuntia robusta* fruit extract displayed strong antioxidant activity linked to its cytoprotective effect [159].

Cancer prevention and treatment is a challenging field that attracts the attention of researchers towards the role of bioactive molecules in the modulation of some immunological and genetic alterations associated with carcinogenesis. Another research study investigated the action of anticin b, a compound extracted from the *Antrodia camphorata* mushroom, on hepatocellular carcinoma cells. It reported that anticin b is a potent apoptosis enhancer and inducer [160].

This carcinogenic opposing effect is interestingly claimed by many researchers to be yielded by some bioactive compounds found in coffee and tea; a positive correlation was found by Lee et al., who analysed data from a Japanese cohort study that declared the reduced risk of colorectal cancer in coffee-consuming Japanese women groups involved in this study [161]. On the other hand, these claims have been investigated in a European Prospective Investigation into Cancer and Nutrition (EPIC) cohort study, and they proposed a relationship between coffee consumption and the reduction of the risk of colorectal cancer. However, this hypothesis was not proved [162]. Taken together, this study showed that coffee and tea consumption is less likely to be associated with the overall CRC risk.

## 10. Conclusions

The human gut microbiota and bioactive compounds hold immense potential in preserving human health and managing inflammatory diseases. The gut microbiota’s complex ecosystem and its interactions with the host play a vital role in maintaining physiological and protective functions against pathogens. Chronic inflammation underlies many human diseases, and current strategies have limitations in controlling it. Bioactive compounds derived from food and plants, such as polyphenols and flavonoids, exhibit anti-inflammatory properties and offer promising therapeutic options. Understanding the role of these compounds and their interplay with the gut microbiota in modulating inflammation is crucial for developing effective treatments. Further research is needed to unravel specific mechanisms and optimise their use in protecting human health against chronic inflammatory disorders.

## Figures and Tables

**Table 1:** Microbiota taxonomy and its relationship with BCs in the regulation of inflammation.

Bioactive Compound	Targeted Microbiota	Effects on Microbiota	Effects on Inflammation	Reference
Polyphenols	*Bifidobacteria*, *Lactobacilli* *Clostridia* *Bifidobacterium* and *Lactobacillus* *Faecalibacterium prausnitzii* *Roseburia* species.	- Increased abundance of beneficial bacteria - Enhances microbial diversity - Reduced pathogenic bacteria	- Downregulation of pro-inflammatory cytokines - Inhibition of NFκB signalling - Suppression of inflammatory responses - Contribute to the gut barrier protection.	[35]
Prebiotics	*Bifidobacteria*, *Lactobacilli*	- Stimulation of growth and activity of beneficial bacteria - Increased SCFAs production	- Attenuation of gut permeability - Reduction of systemic inflammation - Improvement of gut barrier function	[36]
Probiotics	*Lactobacillus* species, and *Bifidobacterium* species	- Introduction of beneficial live bacteria into the gut - Modulation of gut microbial balance - Enhanced production of anti-inflammatory substances	- Reduction of pro-inflammatory cytokines - Regulation of immune responses - Amelioration of inflammation-related disorders	[36]
Resveratrol	*Bacillus* species, *Lactobacillus* species, *Bifidobacterium* species, *Ackermania* species.	- Restore the gut bacteria to its homeostatic levels. - Enhanced growth of beneficial bacteria - Reduced pathogenic bacteria -Increased production of beneficial metabolites	- Attenuated colonic inflammation	[24,37]
Quercetin	*Bifidobacterium* and *Akkermansia*	- Modulate the total microbial population in the gut.	- Anti-inflammatory effects.	[25]
Dietary fibres	*Bifidobacterium* and *Faecalibacterium prausnitzii*	- SCFAs produced through fermentation of dietary fibres by gut bacteria promote the growth of beneficial bacteria - Regulate gut immune responses	- Anti-inflammatory effects on gut epithelial cells - Maintenance of gut barrier function - Attenuation of systemic inflammation	[31,32,38]

## Data Availability

Not applicable.
